# Activity Trackers Implement Different Behavior Change Techniques for Activity, Sleep, and Sedentary Behaviors

**DOI:** 10.2196/ijmr.6685

**Published:** 2017-08-14

**Authors:** Mitch Duncan, Beatrice Murawski, Camille E Short, Amanda L Rebar, Stephanie Schoeppe, Stephanie Alley, Corneel Vandelanotte, Morwenna Kirwan

**Affiliations:** ^1^ School of Medicine & Public Health, Priority Research Centre for Physical Activity and Nutrition Faculty of Health and Medicine The University of Newcastle Callaghan Australia; ^2^ Freemasons Foundation Centre for Men’s Health School of Medicine University of Adelaide Adelaide Australia; ^3^ Physical Activity Research Group School of Medical, Health and Applied Sciences Central Queensland University Rockhampton Australia; ^4^ School of Science and Health Western Sydney University Campbelltown Australia

**Keywords:** health behavior, public health, exercise, sleep, behavior change, fitness trackers, adult, mobile applications

## Abstract

**Background:**

Several studies have examined how the implementation of behavior change techniques (BCTs) varies between different activity trackers. However, activity trackers frequently allow tracking of activity, sleep, and sedentary behaviors; yet, it is unknown how the implementation of BCTs differs between these behaviors.

**Objective:**

The aim of this study was to assess the number and type of BCTs that are implemented by wearable activity trackers (self-monitoring systems) in relation to activity, sleep, and sedentary behaviors and to determine whether the number and type of BCTs differ between behaviors.

**Methods:**

Three self-monitoring systems (Fitbit [Charge HR], Garmin [Vivosmart], and Jawbone [UP3]) were each used for a 1-week period in August 2015. Each self-monitoring system was used by two of the authors (MJD and BM) concurrently. The Coventry, Aberdeen, and London-Refined (CALO-RE) taxonomy was used to assess the implementation of 40 BCTs in relation to activity, sleep, and sedentary behaviors. Discrepancies in ratings were resolved by discussion, and interrater agreement in the number of BCTs implemented was assessed using kappa statistics.

**Results:**

Interrater agreement ranged from 0.64 to 1.00. From a possible range of 40 BCTs, the number of BCTs present for activity ranged from 19 (Garmin) to 33 (Jawbone), from 4 (Garmin) to 29 (Jawbone) for sleep, and 0 (Fitbit) to 10 (Garmin) for sedentary behavior. The average number of BCTs implemented was greatest for activity (n=26) and smaller for sleep (n=14) and sedentary behavior (n=6).

**Conclusions:**

The number and type of BCTs implemented varied between each of the systems and between activity, sleep, and sedentary behaviors. This provides an indication of the potential of these systems to change these behaviors, but the long-term effectiveness of these systems to change activity, sleep, and sedentary behaviors remains unknown.

## Introduction

Higher levels of moderate-to-vigorous intensity activity, lower levels of sedentary behavior, and sufficient sleep on a daily basis are key components of maintaining a healthy lifestyle that is associated with improved quality of life, reduced risk of cardiovascular disease, and diabetes [1-3]. Yet, many adults are not sufficiently active for health benefits, spend considerable amounts of time in sedentary activities, and do not obtain sleep that is of a sufficient duration or quality [4-7]. There are numerous published intervention studies that aim to improve physical activity, sedentary, and sleep behaviors [8-11], and although many are effective, few are disseminated to the broader public [12].

Burgeoning technological innovations mean that mobile devices (smartphone or tablets) and wearable technology such as wrist worn activity trackers, now have increasingly sophisticated capabilities to capture, analyze, and provide feedback to users on their daily physical activity, sleep, and sedentary behaviors. Public interest in this technology is substantial, and adoption of this technology exceeds that of many interventions. Mobile device ownership is increasing, with nearly 80% of people owning a smartphone and 47% owning a tablet [13] and 10% of US adults owning an activity tracker [14]. Studies that critique the potential effectiveness of apps and websites to change behavior conclude that the majority of apps and websites do not contain features or functionality, which are thought to be effective in changing behaviors [15-18]. These critiques have been guided by the availability of behavior change techniques (BCTs) that are potentially effective in changing health behaviors such as goal-setting and self-monitoring [18-21].

The combination of apps, websites, and wearable trackers which synchronize data between them provides a “self-monitoring system,” allowing users to self-monitor their physical activity, sleep, and sedentary behaviors. Despite existing self-monitoring systems providing information on all three behaviors, previous reviews of self-monitoring systems have focused on a single behavior, in most cases physical activity [15,16,18,21-23]. As a result, it is unknown if the approaches implemented by self-monitoring systems to change behavior differ between physical activity, sleep, and sedentary behaviors. In addition, although there is emerging evidence regarding the potential of BCTs to promote behavior change, there is also debate concerning how the number of BCTs and the cooccurrence of BCTs can influence behavior change [20,24-27]. Therefore, examining differences in the number or type of BCTs included in self-monitoring systems for physical activity, sleep, and sedentary behaviors is a first step toward describing the differences in the potential effectiveness of the systems to change these behaviors. Therefore, the purpose of this study was to examine how the number and type of BCTs implemented in self-monitoring systems targeting activity, sleep, and sedentary behaviors differs for each behavior.

## Methods

### Self-Monitoring System Inclusion Criteria and Descriptions

Self-monitoring systems included in this review were the Fitbit Charge HR, Garmin Vivosmart2, and Jawbone UP3 and their respective mobile phone apps and websites. The Fitbit and Jawbone systems were selected for inclusion based on a 2014 review, which indicated that these systems included the highest number of BCTs in relation to physical activity of the 13 systems evaluated [18]. The Garmin system was not included in the prior review but was included in this review as the system includes a “vibration alert.” This feature is also included in the Jawbone and can be used to alert wearers to the fact that they have not taken any steps in the previous hour, which may be useful in assisting wearers to reduce their sedentary behavior. Inclusion criteria were that the self-monitoring systems include a wearable activity tracker that measured physical activity levels, sedentary behavior, and sleep; and an app and/or website that provided the user with information on their behaviors. The activity tracker in all three systems was worn on the wrist. This represents a comprehensive monitoring system. The Jawbone system included an activity tracker and app only and did not include a website that provided feedback to users on their behaviors, whereas the Fitbit and Garmin systems included all three components. This study did not require ethics committee approval, and no informed consent was required as it did not involve participants.

### Coding and Data Extraction

Two trackers for each system were available, so two authors (MJD and BM) could concurrently use each system for a 1-week period. This included wearing the activity tracker and using the app and website (if available). Each author wore the same model of activity tracker, used the same version—the most recent version available at the time of wearing—of the app software on an Apple-based device (mobile phone and tablet), over the same 1-week period. Each activity tracker was worn during all daytime and sleep periods, except for when engaged in water-based activities, if the units were not water proof. At the end of each wear period, the features and content of the systems were independently coded using the Coventry, Aberdeen, and London-Refined (CALO-RE) taxonomy that contains a list of 40 BCTs [19]. The presence or absence of each BCT was coded specifically for the behavior of interest. For coding purposes, physical activity was defined as steps and/or moderate-to-vigorous intensity physical activity; sleep was defined as sleep quality, sleep duration, and/or sleep timing; and sedentary behavior was defined as sitting or standing stationary. This definition of sedentary behavior differs to other definitions which would not classify standing stationary as sedentary [28]; however, this operational definition was necessary as previous experience using the systems showed that standing stationary is classified as sedentary by the systems. For instance, to be coded as allowing users to set goals for sedentary behavior, the system had to allow the user to specifically set goals for that behavior (eg, maximum amount of sedentary behavior performed each day or hour). Agreement between coders on the number of BCTs present for each behavior within each system was calculated using Kappa statistics, and the magnitude of agreement was interpreted using the following criteria: 0.00=poor, 0.01-0.20=slight, 0.21-0.40=fair, 0.41-0.60=moderate, 0.61-0.80=substantial, and 0.81-1.00=almost perfect [29]. The coders then met to discuss any discrepancies in coding, and all discrepancies were resolved to produce a coding summary that is presented in Tables 1 and 2. All use of the systems and coding was conducted in August 2015.

## Results

### Summary of Behavior Change Techniques (BCTs) Implemented

Table 1 summarizes the number of BCTs coded as present for each behavior within each system. The version of the software used for each system is detailed in Table 1 footnotes. Between-rater agreement ranged from 0.64 to 1.00, representing substantial to almost perfect agreement. From a possible range of 40 BCTs, the number of BCTs present for physical activity ranged from 19 (Garmin) to 33 (Jawbone), from 4 (Garmin) to 29 (Jawbone) for sleep, and 0 (Fitbit) to 10 (Garmin) for sedentary behavior. When averaged across systems, self-monitoring systems implemented the highest number of BCTs for physical activity (n=26), a smaller number of BCTs were implemented for sleep (n=14), and the fewest BCTs were implemented for sedentary behavior (n=6). The total number of BCTs included within a system also varied (Table 1). The system that included the highest number of BCTs (n=69) across the three behaviors was Jawbone, followed by Fitbit (n=35), and then Garmin (n=33).

**Table 1:** Summary of the number of behavior change techniques (BCTs) implemented in relation to physical activity, sleep, and sedentary behaviors.

System and behavior	CALO-RE^a^			
	Rater 1 No. BCT^b^	Rater 2 No. BCT	Kappa	Summary No. BCT
Fitbit Charge HR^c^				
Activity	23	27	0.68	25
Sleep	10	12	0.75	10
Sedentary	0	0	1.00	0
Garmin Vivosmart2^d^				
Activity	18	19	0.95	19
Sleep	2	4	0.64	4
Sedentary	10	10	1.00	10
Jawbone Up3^e^				
Activity	33	33	1.00	33
Sleep	29	28	0.82	29
Sedentary	7	7	1.00	7

^a^CALO-RE: Coventry, Aberdeen, and London-Refined.

^b^BCT: behavior change technique.

^c^Fitbit app version 84.

^d^Garmin app version 2.13.1.

^e^Jawbone Up3 app version 4.7.0.121.

### Activity

Table 2 displays which of the 40 BCTs were present within each system for monitoring physical activity. All three systems implemented the following 18 BCTs: providing information about others’ approval, providing normative information about others behavior, goal setting (behavioral and outcome), goal review (behavioral and outcome), prompt rewards contingent on progress toward goal, prompt rewards contingent on successful behavior, shaping, self-monitoring (behavior and outcome), prompting focus on past success, providing feedback on performance, agreeing to behavioral contracts, facilitate social comparison, plan social support, prompt identification of role model, and relapse prevention. Table 2 also details the 6 BCTs that were not implemented in any of the three self-monitoring systems. These were model or demonstrate the behavior, prompt anticipated regret, prompt self-talk, fear arousal, prompt use of imagery, and general communication skills training.

**Figure 1:**
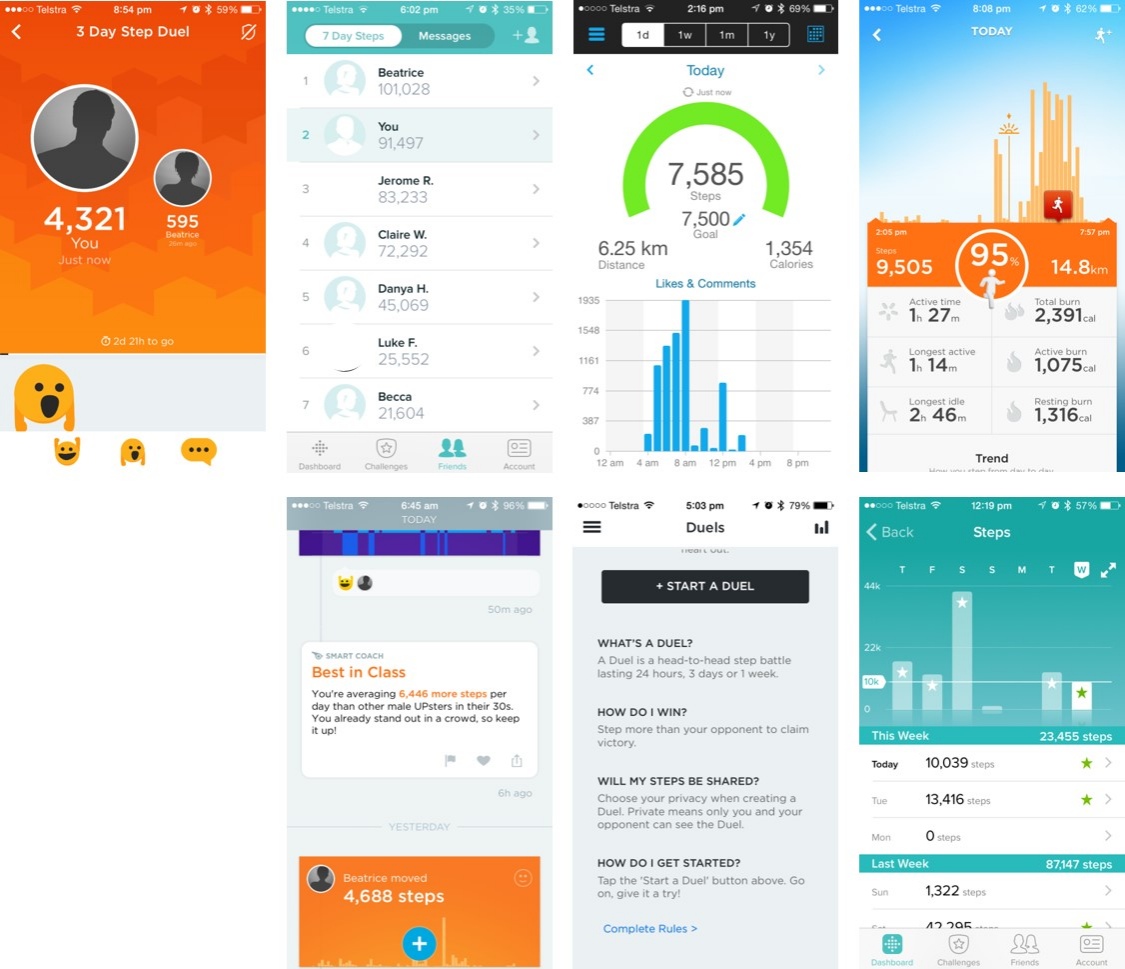
Screenshots of the app or website displaying how various behavior change techniques (BCTs) related to physical activity were implemented.

The activity tracker for all three systems measured physical activity, which was then integrated into the app and/or website to provide users with additional feedback on activity levels. For example, self-monitoring systems frequently implemented BCTs related to social support and social comparisons by allowing peers to offer each other social support through the use of app messaging systems and emoji (Figure 1), peer leader boards (Figure 1), and/or challenges which displayed to users a history (amount or pattern) of their peer’s physical activity (Figure 1). Challenges may offer users a “behavioral contract”; this can be used to prevent relapse, as physical activity is required to be performed over multiple days and plan necessary actions to achieve this (Figure 1). Self-monitoring, goal setting, evaluating activity in relation to goals, providing rewards on past success, progress toward goals (Figure 1), and providing feedback were typically delivered by graphical display of the volume of physical activity performed on a daily basis in comparison to a specified physical activity goal (Figures 1). Feedback to users on achieving a goal was typically highlighted by changing the color or pattern of a progress bar or adding a unique identifying feature to the progress bar (eg, a “star” or textured bar graph). In addition, the activity trackers of all systems vibrated and provided visual feedback to users’ when the daily activity goal was achieved. The Garmin system automatically generated a goal for the user, whereas the Fitbit and Jawbone systems allowed users to set their own goal. The Garmin and Jawbone systems also automatically (Garmin) prompted a user (Jawbone) to increase their activity goal if they reached it consistently.

**Table 2:** Presence of specific behavior change techniques (BCTs) in relation to activity, sleep, and sedentary behavior.

Behavior change technique	Fitbit Charge HR			Garmin Vivosmart			Jawbone UP3			Total		
	Activity	Sleep	Sedentry	Activity	Sleep	Sedentry	Activity	Sleep	Sedentry	Activity	Sleep	Sedentry
Provide information on consequences of behavior in general	0	0	0	0	0	0	1	1	0	1	1	0
Provide information on consequences of behavior to the individual	0	0	0	0	0	0	1	1	0	1	1	0
Provide information about others’ approval	1	0	0	1	0	0	1	1	0	3	1	0
Provide normative information about others’ behavior	1	0	0	1	0	0	1	1	0	3	1	0
Goal-setting (behavior)	1	1	0	1	0	0	1	1	1	3	2	1
Goal-setting (outcome)	1	1	0	1	0	0	1	1	1	3	2	1
Action Planning	1	0	0	0	0	0	1	1	0	2	1	0
Barrier identification or problem solving	0	0	0	0	0	0	1	1	0	1	1	0
Set graded tasks	1	0	0	0	0	0	1	1	0	2	1	0
Prompt review of behavioral goals	1	1	0	1	0	1	1	1	0	3	2	1
Prompt review of outcome goals	1	1	0	1	0	1	1	1	0	3	2	1
Prompt rewards contingent on effort or progress toward behavior	1	0	0	1	0	1	1	1	0	3	1	1
Provide rewards contingent on successful behavior	1	1	0	1	0	1	1	1	0	3	2	1
Shaping	1	0	0	1	0	0	1	0	0	3	0	0
Prompting generalization of a target behavior	0	0	0	0	0	0	1	0	0	1	0	0
Prompt self-monitoring of behavior	1	1	0	1	1	1	1	1	1	3	3	2
Prompt self-monitoring of behavioral outcome	1	1	0	1	1	1	1	1	1	3	3	2
Prompting focus on past success	1	1	0	1	1	1	1	1	0	3	3	1
Provide feedback on performance	1	1	0	1	1	1	1	1	1	3	3	2
Provide information on where and when to perform the behavior	1	0	0	0	0	0	1	1	0	2	1	0
Provide instruction on how to perform the behavior	1	0	0	0	0	0	1	1	0	2	1	0
Model or demonstrate the behavior	0	0	0	0	0	0	0	0	0	0	0	0
Teach to use prompts or cues	1	0	0	0	0	1	1	1	1	2	1	2
Environmental restructuring	0	0	0	0	0	0	0	1	0	0	1	0
Agree behavioral contract	1	0	0	1	0	0	1	0	0	3	0	0
Prompt practice	1	0	0	0	0	1	1	1	1	2	1	2
Use of follow-up prompts	0	0	0	0	0	0	1	1	0	1	1	0
Facilitate social comparison	1	0	0	1	0	0	1	1	0	3	1	0
Plan social support or social change	1	0	0	1	0	0	1	1	0	3	1	0
Prompt identification as role model or position advocate	1	0	0	1	0	0	1	0	0	3	0	0
Prompt anticipated regret	1	0	0	0	0	0	0	0	0	0	0	0
Fear arousal	0	0	0	0	0	0	0	0	0	0	0	0
Prompt self talk	0	0	0	0	0	0	0	0	0	0	0	0
Prompt use of imagery	0	0	0	0	0	0	0	0	0	0	0	0
Relapse prevention or coping planning	1	0	0	1	0	0	1	1	0	3	1	0
Stress management or emotional control training	0	0	0	0	0	0	1	1	0	1	1	0
Motivational interviewing	0	0	0	0	0	0	1	0	0	1	0	0
Time management	0	0	0	0	0	0	1	1	0	1	1	0
General communication skills training	0	0	0	0	0	0	0	0	0	0	0	0
Stimulate anticipation of future rewards	0	1	0	1	0	0	1	1	0	2	2	0
Total	25	10	0	19	4	10	33	29	7	77	43	17

### Sleep

**Figure 2:**
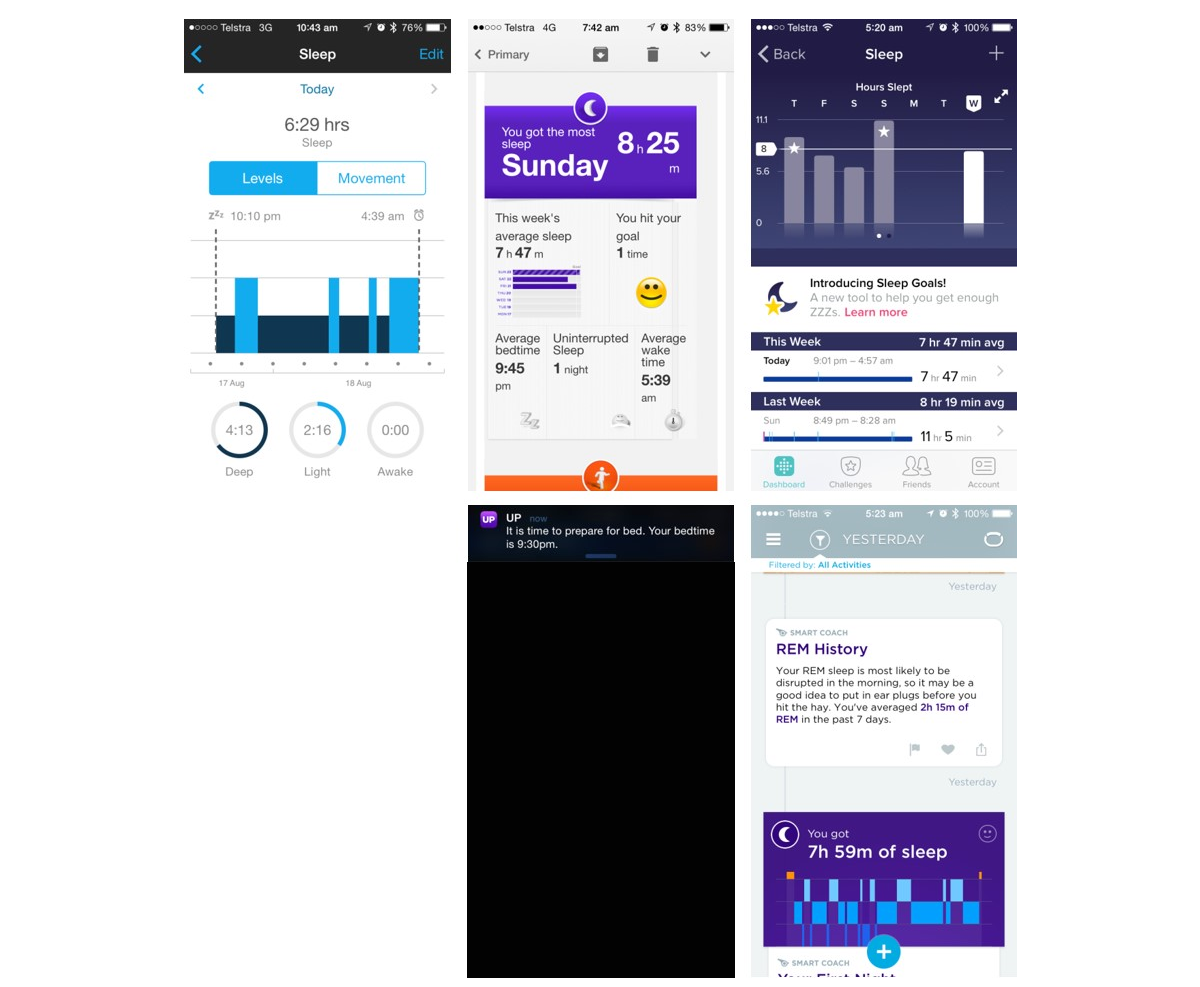
Screenshots of the app or website displaying how various behavior change techniques (BCTs) related to sleep were implemented.

All three systems implemented the following four BCTs: prompt self-monitoring (behavior and outcome), prompt focus on past success, and provide feedback on performance (Table 2). In terms of how these BCTs were implemented in each system, the activity tracker component of all systems provided a measure of sleep volume and quality. This information was then used to generate feedback to users, focus on past success, and providing feedback were implemented by providing graphical display on the volume and quality of sleep (Figure 2). In addition, Fibit and Jawbone systems also implemented the following 6 BCTs: goal setting (behavior and outcome), prompting review of goals (behavior and outcome), providing rewards contingent on successful behavior, and stimulate anticipation of future rewards. These were operationalized by identifying whether the volume and/or quality of sleep (Figure 2) met a user’s goal or not (Figure 2) and by altering the graphical feedback provided by changing the color or pattern of a progress bar or adding a unique identifying feature to the progress bar (eg, a “star” or textured bar graph). The Jawbone system also implemented action planning, prompting, relapse prevention, time management (Figure 2), and environmental restructuring (Figure 2). Table 2 details the 11 BCTs that were not implemented by any of the self-monitoring systems.

### Sedentary Behavior

**Figure 3:**
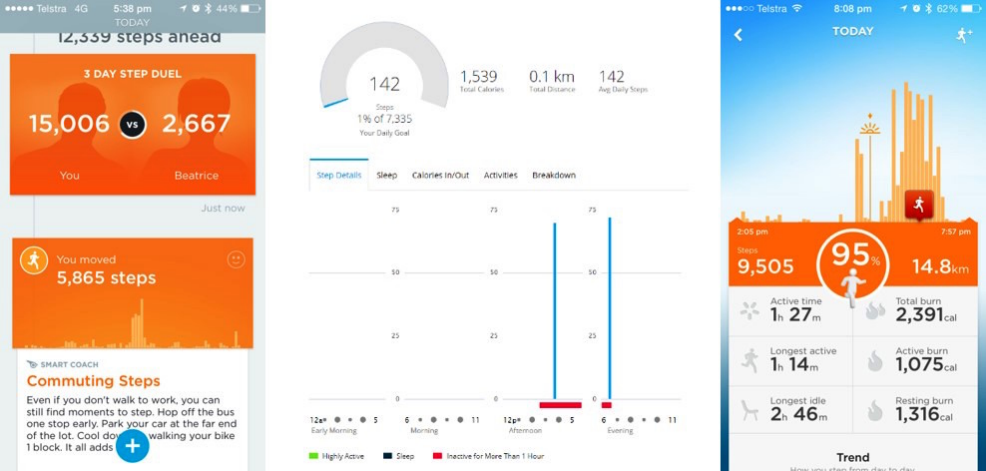
Screenshots of the app or website displaying how various behavior change techniques (BCTs) related to sedentary behaviour were implemented.

The Fitbit system did not implement any BCT in relation to sedentary behavior. The Garmin and Jawbone systems both applied the following five BCTs: prompt self-monitoring (behavior and outcome), provide feedback on performance, teach prompts, and prompt practice. These BCTs were implemented by the activity tracker monitoring periods of no physical activity or steps and then displaying this information to users in terms of the volume of sedentary behavior (Figure 3) and specifically identifying periods of “long” sedentary behavior (Figure 3). Both Garmin and Jawbone units provided feedback to users via the activity tracker, vibrating to indicate if they had been sedentary for a “long” period of time. The Garmin system had a default setting of 1 hour of sedentary activity, which could not be altered by users, whereas the Jawbone system allowed this to be defined by the user. This difference resulted in the Garmin being coded as absent for goal setting (behavioral and outcome) in relation to sedentary behavior, whereas the Jawbone was coded as present. Table 2 displays the 28 BCTs that were not implemented by any of the systems in relation to sedentary behavior.

## Discussion

### Principal Findings

This study assessed the number and type of BCT that three self-monitoring systems implemented to support users in changing their physical activity, sleep, and sedentary behaviors and summarized how the most prevalent BCTs were implemented. The number of BCTs implemented varied between these behaviors. On average, the greatest number of BCTs were implemented in relation to physical activity, followed by sleep and sedentary behavior. All three systems provided self-monitoring of physical activity and sleep and provided feedback to allow the user to focus on their previous success with changing the behavior.

The type of BCT implemented in each system for monitoring physical activity was similar to that observed in other studies [18]. A major difference in the BCT implemented between physical activity, sleep, and sedentary behaviors was the use of challenges, leader boards, and peer to peer “messaging” for physical activity and not for sleep or sedentary behavior. These features operationalize BCTs related to action planning, providing information about others behaviors, social support, shaping, peer approval, and relapse prevention, which may be useful in changing behaviors [19]. These differences may reflect the inherent differences between behaviors and approaches to changing them. For example, the more physical activity people perform, the greater their health benefits [30], and this lends itself to the concept of leader boards and challenges, which can involve frequent peer-to-peer interactions. Yet, for sleep duration, more is not always better as sleep duration has a U-shaped curve in relation to health [31,32], and the concept of “good” sleep is highly individualistic resulting from a complex interaction between the duration, timing, and quality of sleep [1]. As such, whereas goal setting and feedback can be implemented in relation to sleep duration and quality as observed in the systems evaluated (see Figure 2), if leader board and challenge concepts are implemented in relation to sleep, they likely need to be configured around parameters of sleep that are more under the control of the individual, such as sleep hygiene behaviors. For example, the number of days or nights a person went to sleep and woke up at times that “matched” their goals for these behaviors. Alternatively, the concept of leader boards may not be appropriate for sleep. Furthermore, it is important to implement any BCT that seek to improve sleep behaviors in ways which do not increase worry and anxiousness regarding sleep, as this may be detrimental to improving sleep [33].

For sedentary behavior, in light of growing evidence that regular activity breaks are beneficial in comparison with continuous sitting, it may be useful to configure the concept of leader boards and challenges around this premise [34,35]. Leader boards and challenges were implemented in all three systems evaluated in this study in relation to physical activity and are also increasingly implemented in physical activity promotion websites [16]. These features were coded as BCT related to social support, shaping, and relapse prevention; yet, it is unknown how this type of electronic social support compares with in-person peer support and how this influences the efficacy of these strategies. A review of “online social networks” concluded that there was only modest evidence regarding their efficacy to increase physical activity, and continued research is required to clarify their efficacy [36]. Similarly, the evaluated systems implemented “badges” to reward users on their accomplishments, as do many physical activity promotion websites [16]. To date, little is known about how users perceive these features and their effectiveness to change behaviors.

Sleep hygiene education is an effective strategy to improve sleep behaviors in populations with clinical sleep disorders and is also thought to be useful in a public health context to improve the sleep for those people who have sleep complaints but do not have a clinical sleep disorder [37,38]. The Jawbone system implemented the greatest number of BCTs in relation to sleep and did so in a way that was broadly consistent with sleep hygiene guidelines on the timing of sleep, stress reduction, and restructuring the sleep environment to promote sleep [37,38]. It achieved this by measuring sleep and providing feedback on goals using the mobile device notification system to prompt the user to begin getting ready for bed and that their goal time to sleep was approaching. When combined with further education and strategies, these features could help users initiate prebed routines including relaxation techniques to reduce stress and also achieve regularity in the timing of sleep. There is some evidence of the efficacy of these approaches in the literature [37-39], yet, their effectiveness when implemented as part of self-monitoring systems is unknown. These are examples of the BCTs implemented within the Jawbone system that were not implemented within the other systems and highlight how the number and type of BCT implemented vary between the evaluated self-monitoring systems for given behaviors.

Two of the three systems (Jawbone and Garmin) included a vibration alert in the wrist worn activity to alert the user that they had been sedentary for a period of time. This may be a useful prompt to engage in physical activity and reduce sitting time and similar strategies have been implemented as part of ongoing interventions [40]. Although this is an example of a behavioral prompt, it is was not coded as present for goal-setting in the Garmin unit as the user could not set the timing of this feature and therefore adjust their goal. It must also be acknowledged that the two systems that provided feedback on sedentary behavior did so from the perspective of a lack of stepping or movement behavior which does not align with recommended definitions of sedentary behavior [41] and is a function of the technical limitations associated with the activity trackers being worn on the wrist and may have influenced the implementation of BCT for this behavior. Goal-setting is a BCT that is frequently implemented in interventions and is associated with behavior change [8,19,24-26,42]. The Garmin system automatically created a step-based goal for individuals based on the activity level (low, medium, and high) entered when creating a user profile and adjusts the goal based on activity levels the previous day. The Garmin system also used a default 1 hour goal for sedentary behavior, which could not be adjusted by the user, and the inability to adjust this goal was why it was coded as absent for this behavior in the Garmin system. It is unclear how the activity level specified when creating a user profile is translated into a step goal, as is how this automatically created goal relates to existing step-based recommendations [43,44]. The Fitbit and Jawbone systems allowed users to specify their own goals. However, a more useful approach to goal setting for self-monitoring systems may be to provide users with information on the level of behavior for optimum health, information on the goal setting process (eg, promote attainable goals and prompt revision of goals in light of performance), and engage them in the goal setting process (eg, personalized goals) to facilitate users setting goals that move them toward improved health, are attainable, and meaningful to the individual. This approach could be translated to activity, sedentary, and sedentary behaviors in efforts to enhance the way in which goal-setting strategies are implemented.

A total of 18 BCTs were implemented by all three systems in relation to physical activity (Table 2), including those previously associated with increased physical activity, such as providing information on the consequences of the behavior (individual and general), goal setting (behavior and outcome), prompt self-monitoring (behavior and outcome), facilitate social support, prompt practice, and prompt rewards contingent on effort or progress [25,42]. Setting behavioral goals, providing unspecified forms of social support, and adding objects to the environment have been identified as promising BCTs for reducing sedentary time [8]. BCTs related to social support were not present in any of the systems in relation to sedentary behavior and provides an opportunity to expand the capability of the systems to include BCTs that are promising to reduce sedentary time. We are unaware of any previous studies examining BCTs in relation to changes in sleep in either self-monitoring systems or intervention studies; therefore, the insights provided in this study are novel. Although the systems included a number of BCTs which are associated with improved behaviors, to date, there is limited effectiveness surrounding the use of self-monitoring systems to improve these target behaviors [45,46].

A number of BCTs were not implemented in any of the evaluated systems for any of the behaviors (Table 2), and many of these same BCTs are also absent from interventions on other lifestyle behaviors [20,24,26]. Several possibilities may explain this. Designers of self-monitoring systems may simply be unaware of the BCTs literature and implement features guided by the functionality of the system (eg, activity trackers measure amount of movement so systems focus on provided feedback on this), features based on app or website design principles, or features desired by users. Alternatively, omissions of certain BCTs may reflect decisions to implement fewer BCTs as effectively as possible rather than to implement as many as possible in a less effective manner. Furthermore, there is a debate concerning dose-response relationships between the number of BCTs and behavior change and if specific clusters of BCTs are more efficacious than other clusters or if certain BCTs are required to cooccur to maximize potential behavior change [20,24-26]. In light of this, decisions on BCT inclusion and implementation in interventions or self-monitoring systems should be based on addressing the specific behavioral determinants of a behavior. Furthermore, it is unknown how the different combinations of BCTs present in the self-monitoring systems for a specific behavior are related to behavior change. This may also explain differences in the number of BCTs implemented between behaviors, as there is a richer literature on the determinants of physical activity compared with sedentary behavior and sleep [8,47-49]. Furthermore, the mere presence of a BCT does not indicate the way in which it is implemented, which has important implications for behavior change.

### Limitations

Limitations of this study include using a behavior change taxonomy that is directed toward changing physical activity and dietary behaviors to assess sleep and sedentary behaviors. Although this was offset by coding the presence or absence of a BCT specifically to the behavior in question. Furthermore, this study did not assess the features and functionality of the systems in relation to sleep hygiene recommendations which are useful in changing sleep behaviors [37,38]. There are many systems currently available, and it is unknown how systems not included in this study compare on their use and implementation of BCTs. All systems were only used over a 1-week period, which is consistent with previous evaluations [18], and a longer period of use may have resulted in a different user experience resulted in additional BCTs being coded as present. However, there are currently no recommendations regarding how long an intervention or self-monitoring system should be used for before coding.

### Conclusions

In conclusion, the number and type of BCT implemented varied between the evaluated self-monitoring systems and the number and type of BCT varied between activity, sleep, and sedentary behaviors. The greatest number of BCTs was implemented in relation to physical activity, followed by sleep and sedentary behavior. However, the number of BCTs does not reflect how a BCT is implemented and presented to users, or the cooccurrence of a particular BCT with other BCT, which may influence the potential effectiveness of the self-monitoring system to actually change behavior [27]. It is important to note that this study was evaluating the “potential” of these self-monitoring systems to change activity, sleep, and sedentary behaviors and further research is required to establish their effectiveness to change these behaviors. Such evaluations could also examine the actual usage patterns of these devices and the different types of BCTs that users make use of.

## Abbreviations

BCTs: behavior change techniques
CALO-RE: Coventry, Aberdeen, and London-Refined
